# Fabrication of Beta-Barium Borate Sensing Head for Non-Invasive Measurement of Fluidic Concentration Variations

**DOI:** 10.3390/s22249566

**Published:** 2022-12-07

**Authors:** Ruey-Ching Twu, Yi-Ren Sun

**Affiliations:** Department of Electro-Optical Engineering, Southern Taiwan University of Science and Technology, Tainan 71005, Taiwan

**Keywords:** non-invasive technique, heterodyne interferometer, phase interrogation, beta-barium borate

## Abstract

In this study, a beta-barium borate sensing head (BBO-SH) was fabricated and evaluated for the measurements of fluidic concentration variations by using a non-invasive technique. The BBO-SH could be coupled to a fluidic container through thin interlayer water in a heterodyne interferometer based on the phase interrogation. To ensure the sensing head’s stability, the package of BBO-SH uses the prism and the coverslip bounded with UV glue, which can resist environmental damage due to moisture. After each use, the sensing head could be easily cleaned. The sensitivity of the BBO-SH remained stable after repeated measurements over a period of 139 days. Finally, the achievable measurement resolutions of the concentration and refractive index are 52 ppm and 1 × 10^−6^ RIU, respectively, for the sodium chloride solution. The achievable measurement resolutions of the concentration and refractive index were 55 ppm and 8.8 × 10^−7^ RIU, respectively, for the hydrochloric acid solution.

## 1. Introduction

Liquid concentration is an important parameter in the fields of solid immersion lenses [1,2], physiological examinations [3,4], and chemical industry production lines [5]. The measurement of liquid concentration is crucial to the quantitative analysis and synthesis of biomolecules [6]. In order to achieve the desired goal of substance synthesis and effect, it is necessary to develop an accurate and real-time monitoring procedure for concentration variations. Most of the existing schemes are based on measurements of the density [7,8,9,10], conductive [11,12,13], dielectric constant [14,15,16], and refractive index [1,2,3,4] in liquids.

Among these schemes, the conductivity can be detected by a metallic sensing head, the density can be detected using ultrasonic technology [9,10], and the dielectric coefficient or refractive index can be measured by electromagnetic waves. In the measuring process, the electrode sensing head needs to make contact with the liquid sample. As the electromagnetic and ultrasonic waves can propagate in liquid media, the propagation characteristics depend on the liquid composition and concentration. Therefore, suitable transducers have been used for non-invasive techniques.

Electromagnetic waves have different spectral ranges, and the visible wavelength with a low divergence beam is typically used in free-space measurement. The probe lights transmit through a transparent container, where the test sample is placed, then the signal is received by a photodetector [17,18,19] or an image sensor [20,21,22]. To avoid cross contamination, disposable low-cost glass or acrylic containers are used for some liquid samples, such as urine and blood [23,24]. Optical non-invasive measurements can provide a simpler and safer method to isolate the causes of diseases. In industrial production, toxic and volatile solutions need to be stored in closed containers. Moreover, some electrochemical reactions represent safety concern for the operators in some harsh environments [22].

The concentration of a liquid sample has a corresponding linear relationship with the refractive index (RI), and the RI change of liquid may cause the behavior change in the speed, phase, or refracted angle of the light propagation. Therefore, the position shift of the probe light spot caused by concentration variations can be detected by a position sensitivity detector [18,19]. Since high-resolution CCD image detection can provide a finer image contrast and larger detection area, it is also an effective method for high-precision metrology. The measurement of dynamic RI distributions was demonstrated by using a combination of total internal reflection and dual-channel simultaneous phase-shifting interferometry [21]. The two-dimensional phase information for monitoring the dynamic reactions of the electroplating process was adopted in a Mach–Zehnder interferometer [22].

In the near-infrared spectral region, optical fiber sensors were widely demonstrated for the RI measurements according to spectral interrogation based on absorption or interference principles [25,26,27]. The structured fiber transducers with periodic structures and cavity designs in the contact-type schemes require additional manufacturing steps to obtain fine measurement sensitivity. Although the optical fiber measurement system has good integration advantages, its cost is relatively high for the development of disposable sensing units.

In the microwave region, the coupling resonance frequency between two closed conductive transmission lines or microwave resonators can be shifted by relying on the dielectric coefficient of an intermedium [14,15,16]. Therefore, the microwave resonator also provides the advantages of using non-contact for RI measurements. However, the resolution and linear range are limited.

In the terahertz (THz) frequency range [28,29], the THz frequency range corresponds to molecular vibrations or relaxation modes such as those for the hydrogen bond. The technique can be used as a noninvasive measurement through resonance absorption without destroying human tissue.

Nonlinear optics crystal beta-barium borate (BBO) with a high resistance to laser-induced damage is a key component in the existing research on deep-UV lasers, femtosecond laser pulses, and entangled photon pair generation [30,31,32]. The wavelength conversion efficiency depends on the crystal direction between the pumping light and the optical axis of the BBO plate due to the birefringence of BBO. Since BBO is birefringent, there are some commercial products of the polarizer and waveplate used in the ultraviolet-visible range. The time–domain spectroscopy was used to study the anisotropy of optical properties for the application in the THz range [33]. The electro-optical property was applied on the E-filed sensor and Q-switch laser system [34,35]. A previous study successfully demonstrated the use of a BBO plate immersed in grapeseed oil for an optical angle sensor [36].

In this study, BBO was developed as an optically sensing head (SH). The BBO-SH was coupled with a fluidic container (FC) using interlayer water for the non-invasive measurement of fluidic concentration variations. The interlayer water had only a light interconnection between the container and the BBO-SH, and the pure water could be easily cleaned from the glass surface. This concept was similar to the immersion lens technology used in the photolithography process [37]. Unlike general contact-type refractometers, the test liquids were directly placed on the surface of the prism head. Therefore, viscous or volatile liquids were not suitable for this measurement. In our design, the well-designed packaging of BBO-SH ensured no contamination of the test liquid in the fluidic container. The BBO-SH continued to show repeatable measurement performance after 139 days, according to several measurements during this period.

## 2. Measurement Principles

BBO is a uniaxial birefringent crystal. There are only two different refractive indices represented by the extraordinary RI ($n_{e}$) and ordinary RI ($n_{o}$). The RI values for $n_{e}$ and $n_{o}$ are 1.5496 and 1.6672 at a wavelength of 632.8 nm, respectively. A probe light with two orthogonal polarizations (s-wave and *p*-wave) shows different RIs in the birefringent BBO plate. The RI is $n_{e}$ when the polarized light is parallel to the direction of the optical axis ($n_{e}$-axis). It is important to ensure the direction between the $n_{e}$-axis and the s-wave. Before packaging, the BBO plate with the different directions for its $n_{e}$-axis was placed on a rotation stage. The right direction can be determined by comparing the phase variation values under the same incident angle change from 45° to 50° for two directions. According to the theoretical calculation, when the s-wave of the probe light is parallel to the $n_{e}$-axis, the uniaxial birefringent crystal can obtain larger phase variation [38].

**Figure 1:**
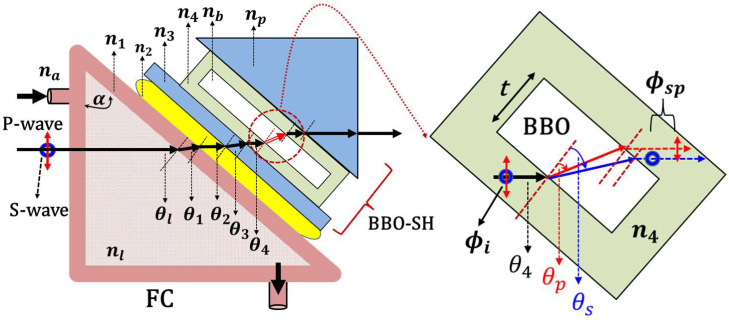
The optical path of the probe light through the FC, the interlayer water, and the BBO-SH.

The optical path of the probe light in the FC coupled with the BBO-SH is illustrated in Figure 1. To avoid the influence of water on the crystal properties of the BBO, it is important to protect the BBO plate ($n_{b})$ with UV glue ($n_{4}$) covered with the coverslip ($n_{3})$ and the prism ($n_{p})$. The protected coverslip and prism can effectively prevent water penetration. Both are made of BK7 glass and have the same RI of 1.515. In this study, a transparent acrylic sheet (*n*1) was used to fabricate the triangular shape FC. The apex angle (α=50°) was used for the FC fabrication. The probe light is normally incident on the front side of the FC; therefore, $\theta_{l}$ is equal to α. The RI of the injection liquid ($n_{l}$) is thus a linear variable depending on the concentration change; this causes the probe light to be deflected slightly through the hypotenuse of the FC. The deflected light passes through the interlayer water ($n_{2}$) and the coupling BBO-SH. The material layers ($n_{l},\;n_{1},\;n_{2},\;n_{3},\;n_{4}$) are isotropic. The propagation angles ($\theta_{1},\;\theta_{2},\;\theta_{3},\;\theta_{4})$ are determined according to Snell’s law in the series of transmission layers, as shown in Equation (1). From the UV glue layer ($n_{4}$) to the BBO plate, the refracted angles $\theta_{s}$ and $\theta_{p}$, as represented in Equations (2) and (3), are for the s-wave and the *p*-wave, respectively. There, $n_{s}$ and $n_{p}$ denote the RIs for the s-wave and *p*-wave, respectively. Based on Equations (1)–(3), the final $\theta_{s}$ and $\theta_{p}$ are obtained through Equations (4) and (5). The refracted angles $\theta_{s}$ and $\theta_{p}$ mainly depend on the values of $n_{l}$ and $\theta_{l}$. The values of $n_{s}$ and $n_{p}$ are 1.5496 and 1.6672 at the wavelength of 632.8 nm, respectively. The subtle angle difference causes different optical paths for both polarizations. The beam overlap between both polarizations causes interference depending on the phase delay from the optical path difference.

Assume the initial phase delay ($\phi_{i})$ between two orthogonal polarizations is equal to zero in the UV glue layer; the final phase delay after passing through the BBO with thickness *t* at probe wavelength λ is expressed as Equation (6). When the probe light has a normal incidence on the front side of the FC, $\theta_{l}$ is equal to α.

(1) $$n_{l}sin\theta_{l}=n_{1}sin\theta_{1}=n_{2}sin\theta_{2}=n_{3}sin\theta_{3}=n_{4}sin\theta_{4}$$

(2) $$n_{4}sin\theta_{4}=n_{s}sin\theta_{s}$$

(3) $$n_{4}sin\theta_{4}=n_{p}sin\theta_{p}$$

(4) $$\theta_{s}=sin^{-1}(n_{l}sin\theta_{l}/n_{s})$$

(5) $$\theta_{p}=sin^{-1}(n_{l}sin\theta_{l}/n_{p})$$

(6) $$\phi_{sp}=\frac{2\pi }{\lambda }t\left(\sqrt{n_{s}^{2}-n_{l}^{2}sin^{2}\theta_{l}}-\sqrt{n_{p}^{2}-n_{l}^{2}sin^{2}\theta_{l}}\;\right)$$

## 3. Non-Invasive Measurement System

**Figure 2:**
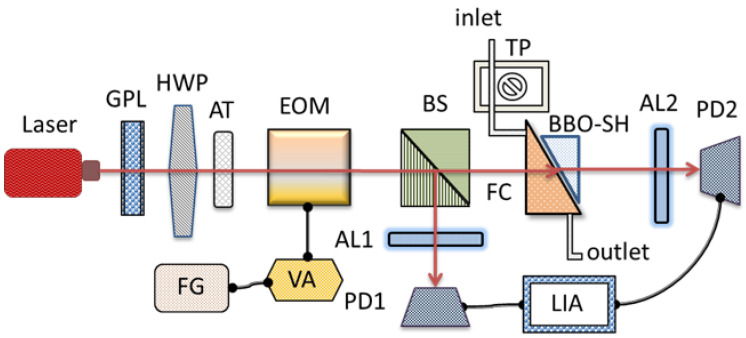
Schematic illustration of the measurement system.

Figure 2 schematically depicts the measurement system utilizing the proposed BBO-SH coupled to the FC in a heterodyne interferometer based on the phase interrogation. A He-Ne laser at 632.8 nm was used as the probe light. A Glan–Taylor polarizer (GPL) and a half wave plate (HWP) aligned at an azimuth angle of 22.5° were used to provide linearly polarized light with two orthogonal polarizations of equal amplitude. An attenuator (AT) was used to control the power of the probe light. The sawtooth-like phase modulation between the two orthogonal polarizations of the probe light was generated by applying an amplified voltage onto an electro-optic modulator (EOM) from a voltage amplifier (VA) connected by a function generator (FG), and the phase-modulated beam was divided into two paths by a beam splitter (BS). The reference signal passed through an analyzer (AL1) to a photodetector (PD1). The BBO-SH coupled onto the FC was placed in the sensing path. The sensing signal passed through an analyzer (AL2) to a photodetector (PD2). Both signals were sent to a lock-in amplifier (LIA) to analyze the variations of the phase delay between the reference and the sensing signals. The different concentrations of liquid were injected into the FC via the inlet port by a tubing pump (TP), and the liquid flowed out through the outlet port. To alternate the exchange of the baseline pure water and different concentrations of the test sample, the switching was operated manually. The variations of the RI or the concentration of the injection liquid could be detected by measuring the phase information through the LIA.

## 4. Results and Discussions

**Figure 3:**
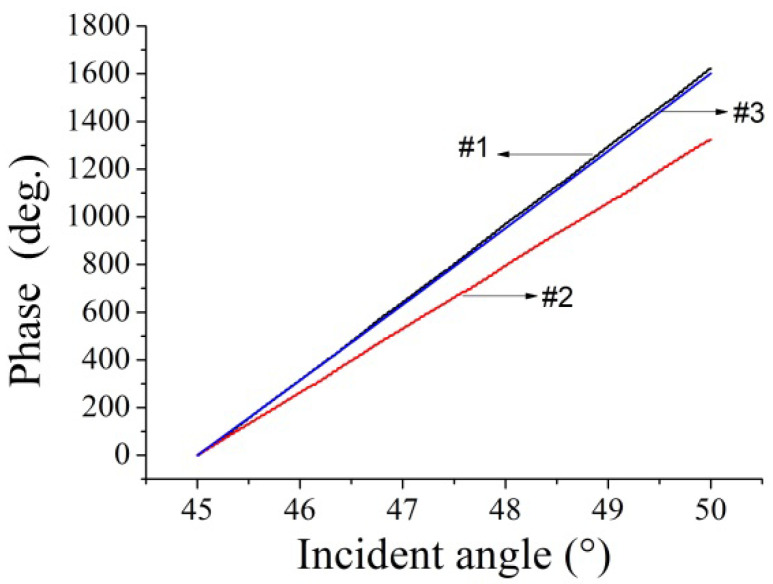
The phase versus the incident angle for the BBO plate scanning angle ranging from 45° to 50°.

Before fabricating the BBO-SH, it was essential to confirm the direction of the BBO optical axis that could achieve higher sensitivity. The evaluation method was performed by measuring the phase variation when the probe light transmitted through the BBO plate (*t* = 1 mm) under an incident angle within the range of 40° to 50°. The BBO was placed on a rotating stage in an air environment. Figure 3 shows the phase measurement results, in which the black (#1) and red (#2) curves represent the s-wave parallel and perpendicular to the $n_{e}$-axis, respectively. Moreover, the simulated blue (#3) curve closely matched the experimental results for the s-wave parallel to the $n_{e}$-axis.

**Figure 4:**
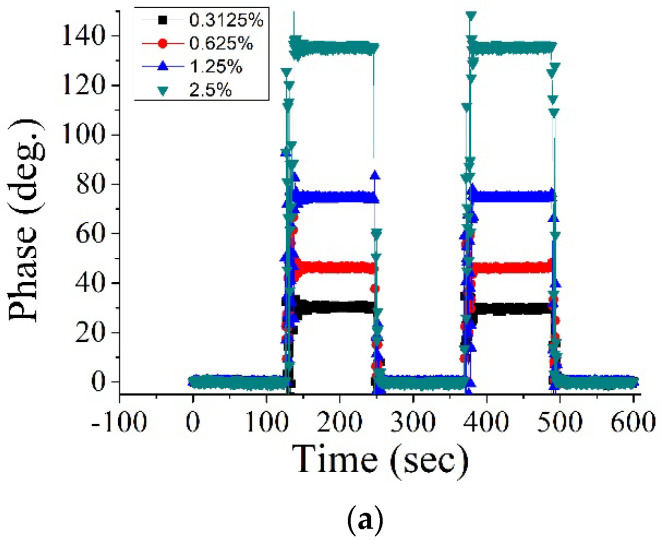

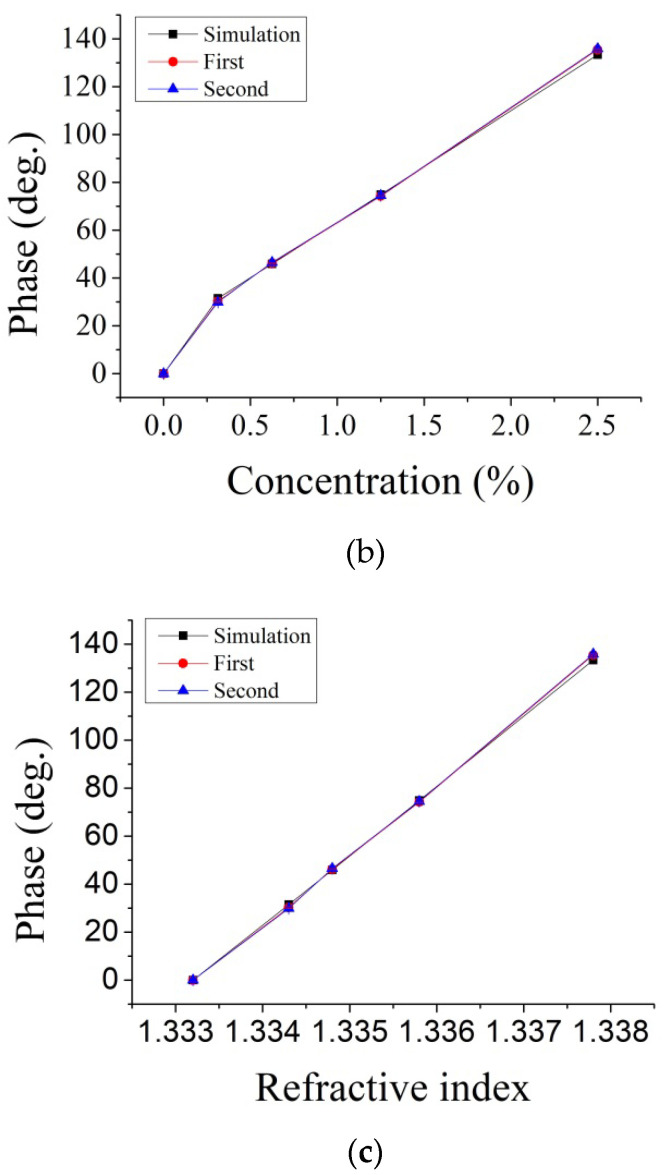
NaCl solution measurement results: (**a**) phase variations by alternately exchanging the pure water and the different NaCl concentrations; (**b**) phase versus concentration for two periods and the simulation results; (**c**) phase versus RI.

The different concentrations of the sodium chloride (NaCl) solution were prepared by mixing pure water and NaCl based on a weight ratio. Figure 4a shows the phase variations for injecting a pulse-like NaCl solution with different concentrations. The mean values of the measured phase by considering the middle period (120 s) were plotted for the phase versus concentration curve, as shown in Figure 4b. The black square, red circle, and blue triangle in Figure 4b denote the first period, secondary period, and simulation results, respectively. According to the measured phase results, the concentration measurement sensitivity (CMS) was defined as a slop value based on a linear fit approach. The RI values of different NaCl concentrations were measured by a commercial refractometer. Then, the plot of the phase versus RI is shown in Figure 4c. The RI measurement sensitivity (RIMS) was defined as a slop value based on a linear fit approach on the phase curves. Based on the analysis results shown in Figure 4b,c, the average values for CMS and RIMS from two periods were 54.28(deg./%) and 2.95 × 10^4^ (deg./RIU), respectively. In this measurement, the standard deviation of the phase stability was 0.28 deg. The achievable measurement resolutions of the concentration and RI were 52 ppm and 1 × 10^−6^ RIU, respectively. The average R-squared values were 0.9825 and 0.9993 for the measurement linearity of the concentration and the RI variations, respectively.

**Figure 5:**
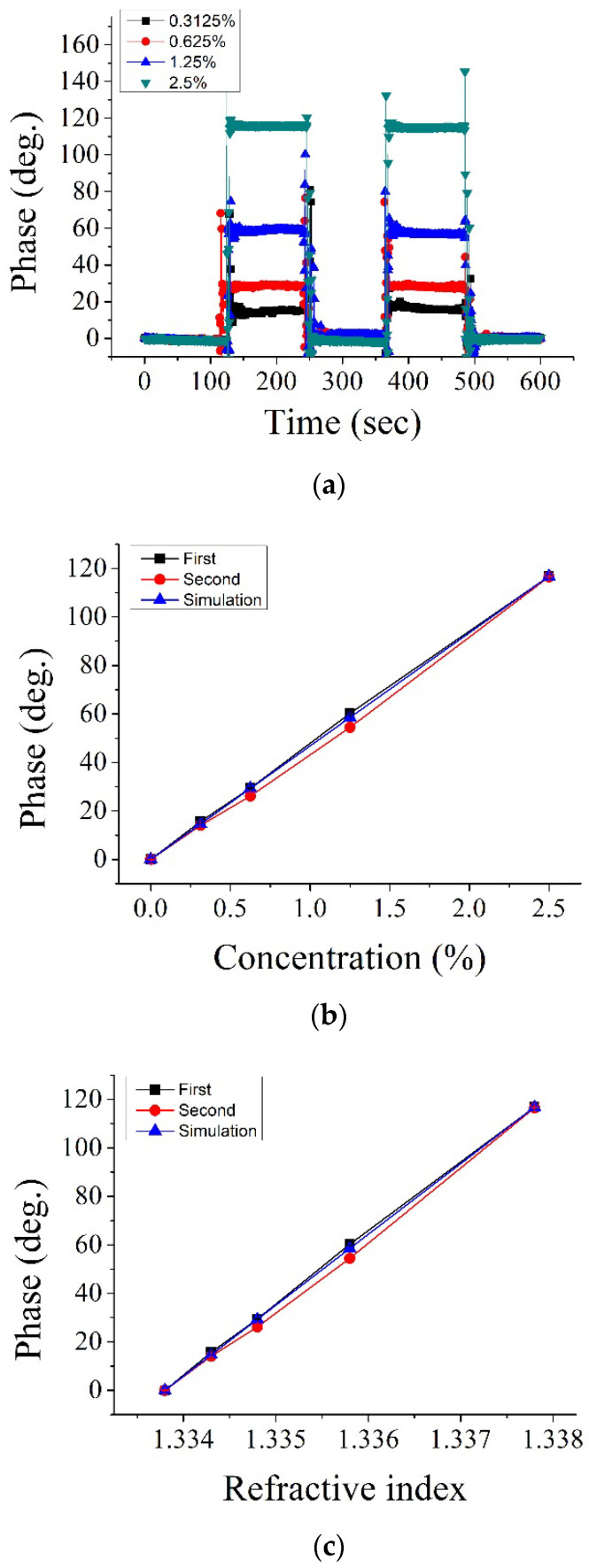
HCl solution measurement results: (**a**) phase variations by alternately exchanging the pure water and the different HCl concentrations; (**b**) phase versus concentration for two periods and the simulation results; (**c**) phase versus the RI.

Optical contact-type refractometers are not suitable for use with corrosive liquids; therefore, metallic sensing heads are often used to detect their conductivity and obtain the concentration change information [5]. In order to demonstrate the non-invasive measurement performance of the proposed methods, the test sample was replaced with different concentrations of hydrochloric acid (HCl) for evaluation. The experimental results are shown in Figure 5. Figure 5a shows the phase variations of injecting a pulse-like HCl solution with different concentrations, Figure 5b shows the phase versus the solution concentration for two periods and the simulation results, and Figure 5c shows the phase versus the solution RI for two periods and the simulation results. Based on the analysis results, as shown in Figure 5b,c, the average CMS and RIMS values were 46.7(deg./%) and 2.92 × 10^4^ (deg./RIU), respectively. In this measurement, the standard deviation of phase stability was 0.26 deg. The achievable measurement resolutions of concentration and RI were 55 ppm and 8.8 × 10^−7^ RIU, respectively. The average R-squared value was 0.9987 for the measurement linearity of both the concentration and RI variations.

**Figure 6:**
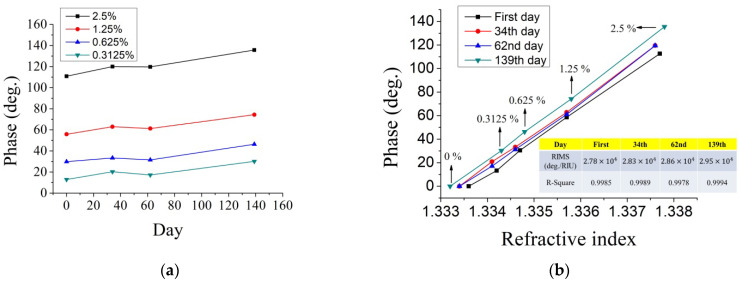
Long-term stability measurement: (**a**) phase versus the measured day for different concentrations; (**b**) phase versus the RI for different measurement days.

To verify the packaged BBO-SH in long-term operation, the measurement conditions were taken by different concentrations of NaCl solutions ranging from 0% to 2.5% each time. After packaging, the measurements were taken on the 1st, 34th, 62nd, and 139th days. Figure 6a shows the phase versus the measured day for different concentrations. The RI values were measured for each experiment. For the phase versus the RI, as shown in Figure 6b, the slope from the linear fit can express the RIMS for each measurement day. Since the RI of the mixed waters was different for the measured day, the initial RI of the pure water (0%) appeared slightly different due to the different room temperatures of the measured day. The summarized table of the RIMS and R-squared values for different days was given in the inset of Figure 6b. Even after a long time, these values remained stable and linear, indicating that the BBO-SH was stable without any damage from moisture and deliquescence.

## 5. Conclusions

This study successfully demonstrated the use of compact BBO-SH for the non-invasive measurement of liquid concentration variations. Although it was necessary to contact the FC through an intermediate water layer, the BBO-SH maintained operational stability after a long-term due to the use of UV glue and covered glass material. At the same time, as the sensing head did not directly contact the liquid in the FC, the fluid could be measured in a confined space. Therefore, the proposed procedure had the advantages of safety and simplicity for the measurement of corrosive or toxic fluids. Moreover, the acrylic FC is easy to manufacture, disposable, and low in cost. The corrosive fluid was verified by measuring the concentration variation of hydrochloric acid fluid, and the results confirmed that the detection resolution can be reached precisely. Finally, the achievable resolutions, concentration, and RI measurements were 55 ppm and 8.8 × 10^−7^ RIU, respectively. The resolution of the present sensing method is better in comparison with other reported techniques [17,18,19,20,21,22].

## Data Availability

Not applicable.
